# Evaluating Oxygen Tensions Related to Bone Marrow and Matrix for MSC Differentiation in 2D and 3D Biomimetic Lamellar Scaffolds

**DOI:** 10.3390/ijms22084010

**Published:** 2021-04-13

**Authors:** Esen Sayin, Erkan Türker Baran, Ahmed Elsheikh, Vivek Mudera, Umber Cheema, Vasif Hasirci

**Affiliations:** 1 Department of Biotechnology, Middle East Technical University, 06800 Ankara, Turkey; esensyn@gmail.com; 2 Department of Tissue Engineering, University of Health Sciences, 34668 Istanbul, Turkey; erkanturkerbaran@gmail.com; 3 School of Engineering, The University of Liverpool, Liverpool L69 3GH, UK; Ahmed.Elsheikh@liverpool.ac.uk; 4 UCL Centre for 3D Models of Health and Disease, Division of Surgery and Interventional Science, University College London, 43-45 Foley Street, Fitzrovia, London W1W 7TY, UK; v.mudera@ucl.ac.uk (V.M.); u.cheema@ucl.ac.uk (U.C.); 5 Department of Medical Engineering, Acibadem Mehmet Ali Aydinlar University, 34752 Istanbul, Turkey

**Keywords:** oxygen tension, 2D vs. 3D, mesenchymal stem cell, osteogenesis, bone tissue engineering

## Abstract

The physiological O_2_ microenvironment of mesenchymal stem cells (MSCs) and osteoblasts and the dimensionality of a substrate are known to be important in regulating cell phenotype and function. By providing the physiologically normoxic environments of bone marrow (5%) and matrix (12%), we assessed their potential to maintain stemness, induce osteogenic differentiation, and enhance the material properties in the micropatterned collagen/silk fibroin scaffolds that were produced in 2D or 3D. Expression of osterix (OSX) and vascular endothelial growth factor A (VEGFA) was significantly enhanced in the 3D scaffold in all oxygen environments. At 21% O_2_, OSX and VEGFA expressions in the 3D scaffold were respectively 13,200 and 270 times higher than those of the 2D scaffold. Markers for assessing stemness were significantly more pronounced on tissue culture polystyrene and 2D scaffold incubated at 5% O_2_. At 21% O_2_, we measured significant increases in ultimate tensile strength (*p* < 0.0001) and Young’s modulus (*p* = 0.003) of the 3D scaffold compared to the 2D scaffold, whilst 5% O_2_ hindered the positive effect of cell seeding on tensile strength. In conclusion, we demonstrated that the 3D culture of MSCs in collagen/silk fibroin scaffolds provided biomimetic cues for bone progenitor cells toward differentiation and enhanced the tensile mechanical properties.

## 1. Introduction

Culturing cells in 3D is typically conducted at atmospheric O_2_, which is approximately 21% [1,2,3,4]. However, in in vivo tissues, including bone, the physiological O_2_ level is much lower between 5–14%, and resident cells respond to the changing O_2_ environment through the hypoxia inducible factor (HIF)-regulated O_2_-sensing mechanism [5]. The bone matrix has ambient O_2_ tension of approximately 12% owing to arterial blood vasculature. This 12% O_2_ level plays a role in maintaining osteoblast phenotype [6], as low O_2_ condition is associated with the stem cell niche and preservation of cells in an undifferentiated state [7]. Unlike the osteoblasts, bone marrow mesenchymal stem cells (MSCs) reside in the sinusoidal cavity. Within this bone compartment, the range of O_2_ availability drops to between 1–6% [8]. As previously proposed by another research group, finding the optimum level of O_2_ tension for the pretreatment of bone tissue-engineered scaffolds can be the key to enhance their functionality through improved osteogenesis [9]. Contrary to the majority of studies using 21% O_2_ to culture MSCs, fewer studies focus on utilizing hypoxia for bone cell culture. Nevertheless, these works on hypoxia do not include scaffolds that are proposed to be used for bone tissue engineering (TE) purposes, but they were about in vitro osteogenic differentiation of MSCs on tissue culture plate in the range of <0.02–5% O_2_ tensions [10,11,12,13].

Studies on the effect of O_2_ tension on the osteogenic activity of various stem cell types have been carried out with tissue culture plates, micromass culture, or 3D environment [10,11,14,15,16,17]. It is established that in vitro 3D culture promotes the osteogenic differentiation potential of adipose-derived stem cells (ADSCs) [15], osteoblasts [18], embryonic stem cells [17], and periodontal ligament fibroblasts [19]. Thus, for more comprehensive results than the data acquired separately, we have evaluated the simultaneous effect of O_2_ tension and 3D culture using biomimetic scaffolds on the stem cell status of MSCs.

The 3D environment influences the osteogenic differentiation of ADSCs both in hypoxic and normoxic environments [15]. For studies directly comparing 2D and 3D culture, cells are often cultured on tissue culture plastic to represent the 2D environment. However, dissecting out the specific effects of either scaffold material or 3D culture is important. Therefore, in this study, the same material was used for 2D and 3D. Dimensionality was therefore specifically under test.

The objective of this research was to concurrently study the influence of O_2_ tension and dimensionality by using the same material in MSC-seeded scaffolds with a motive to improve osteogenic differentiation, angiogenesis, and tensile properties of the cell-seeded scaffold. A blend of collagen type I and silk fibroin was formulated to generate a composite material with flexibility and high tensile strength. We processed this composite in the form of microchannel (MC)-patterned films. Here, parallel MCs were used as a tool to promote the anisotropic extracellular matrix (ECM) secretion as in the aligned natural collagen fibers of bone ultrastructure [20,21]. The first film type acted to recapitulate the bone lamella on a much bigger scale, while the rolled longer film formed an osteon-like 3D scaffold including a canal in the core as in original structure. Thus, we fabricated 2D and 3D scaffolds using the compositional and architectural biomimicry to bone. Then, we tested the physiological O_2_ tensions of bone marrow (5%) and bone matrix (12%) and compared these with the atmospheric in vitro test conditions of ambient O_2_ (21%), which is a standard practice in bone TE.

## 2. Results

### 2.1. Specifications of the 2D and 3D Scaffolds 

Spiral winding technique was used for creating 3D scaffold from microchannel-patterned long film strip. Thus, wetting the scaffolds was necessary for easy of manipulation of films, which were brittle in a dry state. In cell culture, the brittle structure of the dry films changed due to swelling of the silk fibroin and mostly the collagen in the blend [22]. Additionally, crosslinking with 1-ethyl-3-(3-dimethylamino-propyl) carbodiimide hydrochloride/N-hydroxysulfosuccinimide (EDC/NHS) was necessary for optimum scaffold stiffness that enabled straightforward bending in the wet state. That means the EDC/NHS crosslinking procedure allowed for easy rolling of the scaffold, as well as keeping structural integrity upon swelling. We have previously shown that the EDC/NHS methodology did not cause any surface/topography modification and helped the preservation of the micropatterns even after 28 days of cell culture [21].

**Figure 1:**
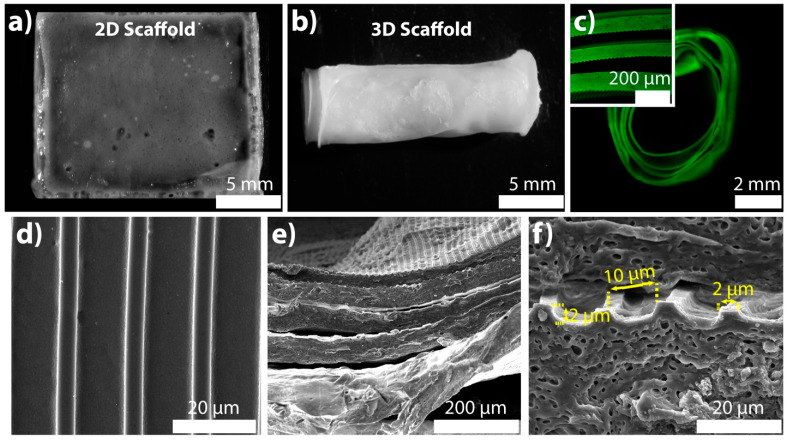
For the physical characterization of the 2D and 3D collagen/silk fibroin scaffolds: stereomicroscope images were taken in the wet state for the (**a**) 2D (top view) and (**b**) 3D (side view) scaffolds. (**c**) Fluorescence microscopy and confocal laser scanning microscopy (CLSM) images to view the cross-section of the 3D scaffold after fluorescein labeling. SEM imaging of (**d**) 2D (scale bar: 20 μm) and (**e**) 3D scaffolds (scale bar: 200 μm). (**f**) SEM image of 3D scaffold’s cross-section (scale bar: 20 μm). The markings on the image indicated the micropattern dimensions.

The thickness of the films was measured after letting them swell in the cell culture medium. The 2D scaffold was 73 ± 12 μm, and the long film strip was 73 ± 15 μm in average thickness. The similar thickness values showed that 2D and 3D scaffolds were consistent in thickness. The 2D rectangular scaffold size was 1.5 cm in width and 1.7 cm in length (Figure 1a). The long film had the same width as the 2D scaffold, and it was 5.7 cm in length. After rolling, the diameter and the height of the 3D scaffold were 0.5 cm and 1.5 cm, respectively (Figure 1b). In cortical bone ultrastructure, osteons are 1–2 cm long and 1–2 mm wide [23]. Although the 3D scaffold was not comparable to the in vivo osteon in size, our aim was to mimic the concentrically layered osteon on a much bigger scale and observe the dimensionality effect.

The 3D scaffold was labeled with fluorescein (green) to observe the layers within the structure. We examined the proximity of the hydrated layers to ascertain and confirm cell contact to both layers. As seen by the fluorescence microscopy image, the rolled 3D scaffold had five full turn layers (Figure 1c), which was biomimetic of the native osteons that are from 5 to 20 concentric lamella layers [24].

The pore size of the 3D scaffolds is an important factor in maintaining a favorable MSC microenvironment by allowing nutrient and waste exchange. To introduce porosity, different techniques such as salt leaching have been used for bulk polymer materials. In a recent study, the porogen size of 75–500 μm was shown to be sufficient to sustain bone marrow MSC viability and osteogenic commitment [25]. The distance between the layers of the 3D scaffold were in the range of 69 μm and 101 μm (Figure 1c, inset). Therefore, the proximity of the layers (77 ± 9 μm) was suitable for forming a 3D microenvironment for MSCs.

MCs on the dry 2D and 3D scaffolds were examined with a scanning electron microscopy (SEM). Patterns on 2D and 3D scaffolds were satisfactory to continue with the in vitro cell culture tests owing to their uniform size (Figure 1c–e). The MC-patterns that evenly distributed on both scaffolds showed the potential to maintain the MSC alignment throughout the scaffolds. The size of the topographical cues was measured using ImageJ software. The ridge’s width and height, and the groove’s width were 2, 2, and 10 μm, respectively (Figure 1f).

### 2.2. Screening the Oxygen Percentage in 3D Scaffold

**Figure 2:**
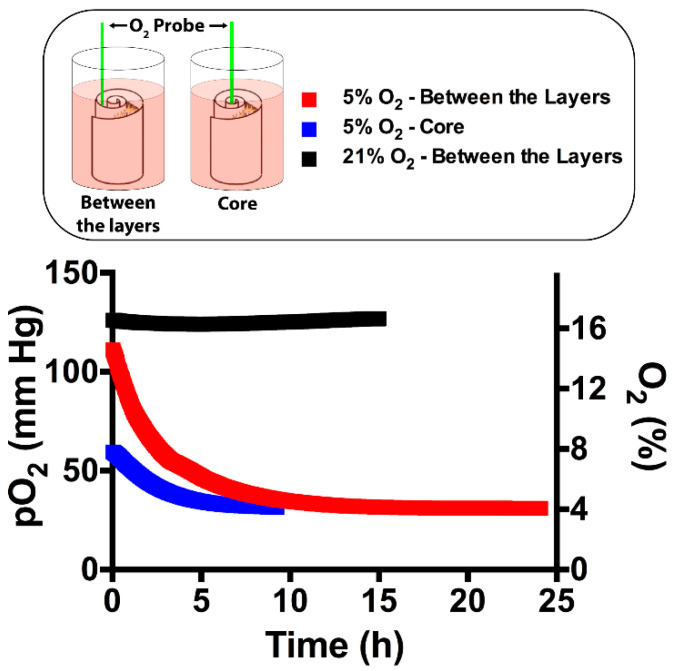
Partial pressure of the O_2_ (pO_2_) inside the mesenchymal stem cell (MSC)-seeded 3D scaffold was determined at 5% and 21% O_2_ tensions. An O_2_ probe was inserted between the layers and into the core. After MSC seeding, O_2_ monitoring was started and recorded until stationary pO_2_ readings.

To measure the partial pressure of the O_2_ (pO_2_), the fiber-optic O_2_ probe was inserted into the core and between the layers of the 3D scaffold (Figure 2). These two regions were selected for data recording to detect the actual concentration that deep cells are exposed to since diffusion can be impaired at the inner sections of a tissue-engineered 3D scaffold. The O_2_ inside the incubator was separately set to 5% and 21% to observe the influence of the most distinct O_2_ tensions. At 21% O_2_, the core level remained constant without any decrease in time as it was in between the layers (Figure 2). At both measurements, the readings were in the range of 124–127 mm Hg, which corresponds to 16.3–16.7%. Thus, the steady O_2_ levels without any sudden drop showed that the environment in the 3D scaffold was suitable in terms of oxygenation. Noticeably, all of the readings were around the pO_2_ of the cell culture medium (approximately 130 mm Hg) [26]. This value was less than the ambient O_2_ level (160 mm Hg) as reported previously due to the limited solubility of O_2_ in the cell culture medium. At 5% O_2_, the pO_2_ at the core and between the layers decreased continuously and reached an equilibrium around 4% (31 mm Hg). As in 21% O_2_, limited O_2_ solubility and no abrupt decrease were noted.

### 2.3. Surface Characterization and Morphology of MSCs 

**Figure 3:**
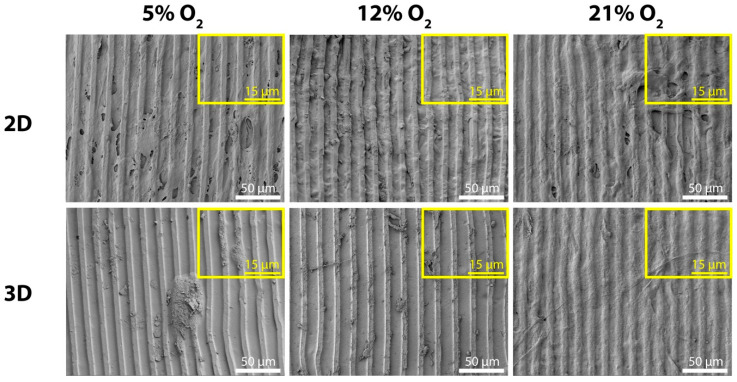
SEM micrographs at day 3 were taken to examine the topography conservation and MSC morphologies on the 2D and the 3D scaffold surfaces (scale bar: 10 μm).

We studied the influence of 5% and 12% O_2_ tension in MSC-seeded 2D and 3D collagen/silk fibroin scaffolds. The shape of MCs was stable, as SEM images demonstrated at day 3 (Figure 3). On the 2D scaffolds, MSCs formed a layer of cells on the ridges and in the grooves of MCs at all O_2_ tensions. Cells were also guided along the MCs on collagen/silk fibroin 2D scaffold surfaces after 3 days of culture. A similar guided cell layer was observed at 21% O_2_ tension on the 3D scaffold surface. However, the behavior of MSCs in 3D had deviations at the rest of the O_2_ tensions. MSCs were individually present in the micropattern grooves at 12% O_2_ tension, and cell clumps existed at 5% O_2_ tension.

### 2.4. Relative Expression of Osteogenic, Stemness, and Angiogenic Markers

To compare the degree of osteogenic differentiation, stemness, and angiogenesis, expression levels of runt-related transcription factor 2 (RUNX2), osterix (OSX), bone marrow stromal cell antigen 1 (BST1), cluster of differentiation 90 (CD90), and vascular endothelial growth factor A (VEGFA) were determined at day 3. Tissue culture polystyrene (TCPS) at 21% O_2_ was selected as a control material for relative gene expression.

**Figure 4:**
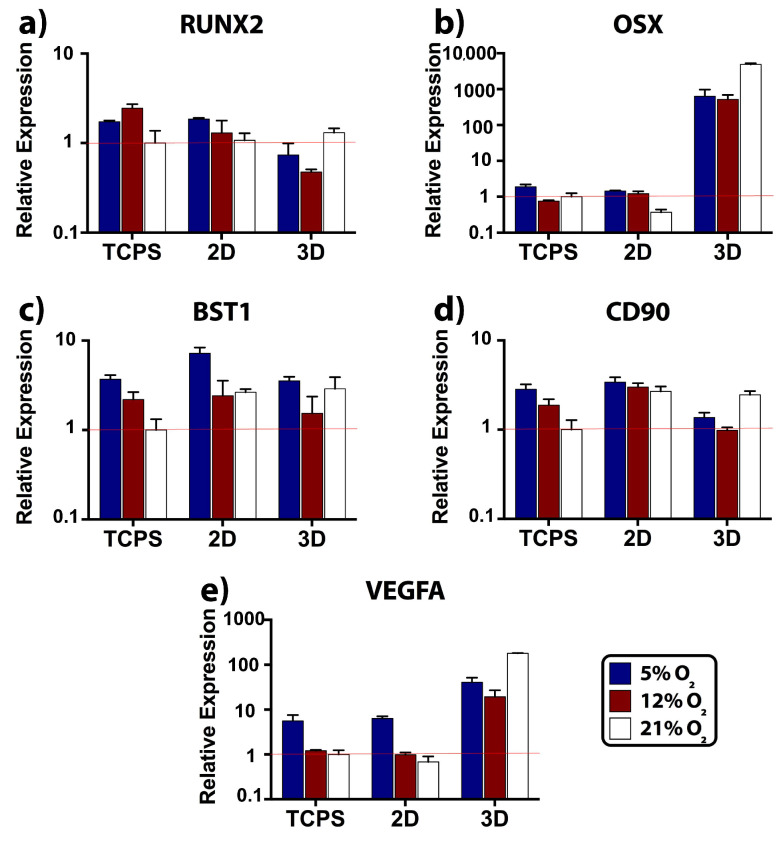
Markers of osteogenic differentiation, stemness, and angiogenic factor were evaluated after 3 days of incubation at 5%, 12%, and 21% O_2_ tensions. Transcript levels on TCPS, 2D scaffold, and in 3D scaffold were determined with real-time reverse-transcription polymerase chain reaction (real-time RT-PCR). Fine red lines indicate the normalized gene expression level, which is designated as 1. Osteogenic differentiation was studied with (**a**) runt-related transcription factor 2 (RUNX2) and (**b**) osterix (OSX). Stemness was examined with (**c**) bone marrow stromal cell antigen 1 (BST1) and (**d**) cluster of differentiation 90 (CD90). (**e**) Angiogenic activity was studied with vascular endothelial growth factor A (VEGFA).

We employed RUNX2 and OSX markers for analyzing the level of osteogenic differentiation. The transformation of MSCs to osteoblasts requires the activation of a molecular switch: RUNX2 [27]. In our study, RUNX2 expression by MSCs was both substrate and O_2_ specific (Figure 4a). The maximum RUNX2 levels were observed at 12% O_2_ on TCPS, at 5% O_2_ on 2D scaffold, and at 21% O_2_ in 3D scaffold. The statistical analysis showed that at 5% and 12% O_2_, RUNX2 expression on TCPS and 2D was significantly higher when compared to 3D (Supplementary Table S1). Meanwhile, RUNX2 expression in 3D was significantly upregulated by three-fold at 21% O_2_ compared to 12% O_2_. Downstream of RUNX2, OSX is regulated, and it enhances collagen type I production [28]. When we evaluated OSX expression we again found this to be substrate and O_2_ specific. At 5% O_2_, both the TCPS and 2D scaffold showed increased OSX expression by MSCs (Figure 4b). At 21% O_2_, OSX was downregulated by three-fold on 2D compared to TCPS. Notably, the 3D scaffold led to an upregulation of OSX at all O_2_ tensions. The ratios of 3D_OSX_ /2D_OSX_ were 442 and 424 at 5% (*p_2D–3D_ =* 0.009) and 12% (*p_2D–3D_ =* 0.019), respectively (Supplementary Table S2). At the 21% O_2_, OSX was expressed 13,200 times more than 2D scaffold, which corresponded to a significant difference (*p_2D–3D_* < 0.0001). At the same O_2_ tension, OSX expression in the 3D scaffold was eight- and nine-fold higher than 5% (*p*_5–21%_ < 0.0001) and 12% (*p*_12%-*21%*_ < 0.0001) O_2_ tensions, respectively. Hence, 12% O_2_ led to the lowest RUNX2 and OSX expressions in the 3D scaffold. Briefly, MSCs in the 3D scaffold differentiated more than those on the TCPS and 2D scaffold.

We used BST1 (Figure 4c) and CD90 (Figure 4d) as the indicators of MSC phenotype and observed the hypoxia-driven stemness on TCPS and 2D scaffold by looking into their levels. BST1 on TCPS (*p*_5–21%_ = 0.009) and 2D scaffold (*p*_5%-*12%*_ < 0.0001, *p*_5–21%_ < 0.0001) was significantly upregulated at 5% (Supplementary Table S3). Likewise, CD90 on TCPS was significantly upregulated at 5% (Supplementary Table S4, *p*_5%-*12%*_ = 0.006, *p*_5–21%_ < 0.0001). We evidently showed the dissimilarities between the TCPS and the 2D scaffold thanks to the statistical differences in BST1 and CD90 expression levels noted in each O_2_ tension (Supplementary Tables S3 and S4). Lower stemness levels in a gene expression study can point out stem cell differentiation. BST1 [29] and CD90 [30] were previously documented to be downregulated as the MSCs proceeded toward osteogenic differentiation. As we anticipated, the expression of stemness markers in the 3D scaffold was significantly lower than on 2D scaffold. To be more specific, the ratios of 3D_CD90_/2D_CD90_ were 2 and 3 at 5% (*p_2D–3D_* < 0.0001) and 12% (*p_2D–3D_* < 0.0001) O_2_ tensions, respectively. Additionally, the ratio of 3D_BST1_/2D_BST1_ was 2 at 5% (*p_2D–3D_* = 0.002). However, stemness levels were close between 2D and 3D scaffolds at 21%, and moreover, at the same O_2_ tension, CD90*_3D_* expression was significantly higher than 5% (*p*_5–21%_ = 0.005) and 12% (*p*_12%-*21%*_ < 0.0001).

Low O_2_ tension activates hypoxia-inducible factor (HIF) secretion by regional osteoblasts during the skeletal repair [31]. Then, HIF acts as a key transcriptional regulator for neovessel formation, and it targets VEGF gene [32]. Similarly, low O_2_ tension was reported to activate HIF and VEGF expressions in the MSC culture [33]. In our study, VEGFA expressions on TCPS control and 2D scaffold were very close to each other, and the levels were higher at 5% O_2_ tension, as expected (Figure 4e). When 3D scaffold was compared with 2D scaffold, we observed the significant positive effect of 3D on VEGFA expression at all O_2_ tensions (Supplementary Table S5). The ratios of 3D_VEGFA_/2D_VEGFA_ were 6, 20, and, 266 at 5% (*p_2D–3D_* < 0.0001), 12% (*p_2D–3D_* = 0.001), and 21% (*p_2D–3D_* < 0.0001) O_2_ tensions, respectively. Strikingly, the VEGFA levels in 3D showed that the 21% O_2_ significantly promoted VEGFA expression (*p*_5–21%_ < 0.0001, *p*_12%-*21%*_ < 0.0001), while the 5% O_2_ led to a statistically higher VEGFA expression than 12% O_2_ (*p*_5%-*12%*_ < 0.0001).

### 2.5. Tensile Properties of Unseeded and MSC-Seeded Scaffolds

**Figure 5:**
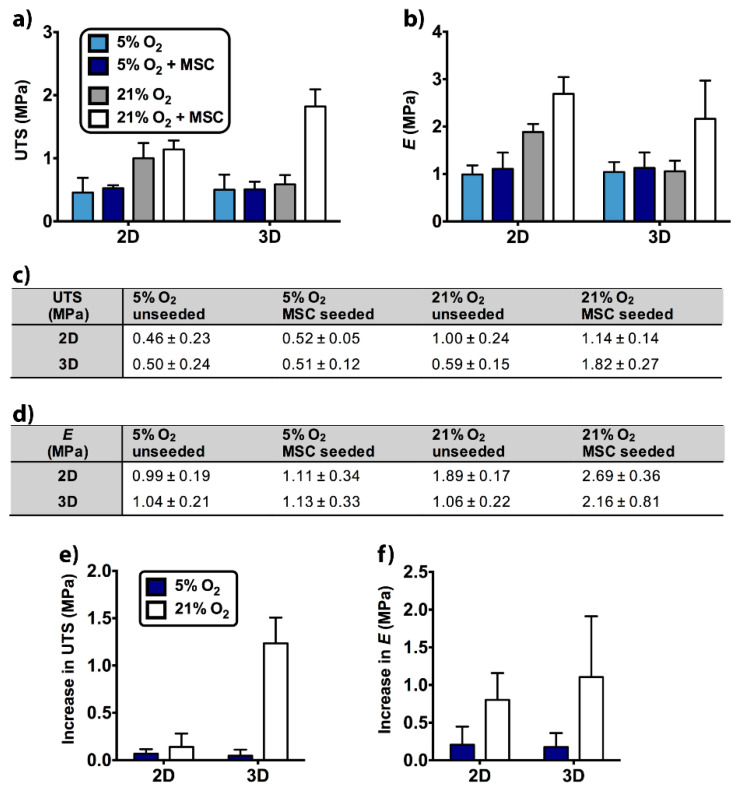
Tensile properties of the 2D and the 3D scaffolds were determined after 35 days of incubation at 5% and 21% O_2_ tensions. Unseeded and MSC-seeded samples were tested in the direction that was parallel to the microchannel (MC) axis. (**a**) Ultimate tensile strength (UTS) and (**b**) Young’s modulus (*E*) were presented as graphs. The average ± standard deviation values of (**c**) UTS and (**d**) *E* were given in tables. (**e**) Increase in UTS and (**f**) increase in *E* were also calculated.

Given the effects of dimensionality and O_2_ tension on the MSC differentiation, the quality of the secreted ECM was evaluated through tensile testing after 35 days of cell culture. At this point, we selected 5% and 21% O_2_ tensions in testing since MSCs exhibited enhanced osteogenic differentiation at these O_2_ tensions on 2D and in 3D scaffolds, respectively. Notably, the level of O_2_ led to significant differences in ultimate tensile strength (UTS) and Young’s modulus (*E*) of the unseeded 2D scaffold (Supplementary Tables S6 and S7). The UTS (Figure 5a,c) and the *E* (Figure 5b,d) values of unseeded 2D scaffold were reported as UTS_5%_ = 0.46 MPa, UTS_21%_ = 1 MPa, *E*_5%_ = 0.99 MPa, and *E*_21%_ = 1.89 MPa. These UTS and *E* values showed that after incubation at 5% O_2_, both UTS (*p*_5–21%_ = 0.008) and *E* (*p*_5–21%_ = 0.019) were significantly lower than those incubated at 21% O_2_.

The significant differences between MSC-seeded scaffolds were also determined. At 21% O_2_, UTS (*p*_5–21%_ = 0.005) and *E* (*p*_5–21%_ < 0.0001) of the 2D scaffold were approximately two-fold higher than 5% O_2_ (UTS_5%_ = 0.52 MPa, UTS_21%_ = 1.14 MPa, *E*_5%_ = 1.11 MPa, and *E*_21%_ = 2.69 MPa). At 21% O_2_, UTS (*p*_5–21%_ < 0.0001) and *E* (*p*_5–21%_ = 0.005) of the 3D scaffold were respectively ~four-fold and ~two-fold higher than 5% O_2_ (UTS_5%_ = 0.51 MPa, UTS_21%_ = 1.82 MPa, *E*_5%_ = 1.13 MPa, and *E*_21%_ = 2.16 MPa). Additionally, dimensionality led to a statistically significant difference in the UTS of MSC-seeded scaffolds. 3D scaffold had a higher UTS value than 2D scaffold at 21% O_2_ (*p_2D–3D_* = 0.002).

When the cell-seeded scaffolds were compared with the unseeded scaffolds, *E* of 2D scaffold showed a statistical difference at 21% O_2_ (*p_unseeded-seeded_* = 0.033). Additionally, UTS (*p_unseeded-seeded_* < 0.0001) and *E* (*p_unseeded-seeded_* = 0.003) showed significant differences in 3D scaffold at 21% O_2_ (UTS_unseeded, 21%_ = 0.59 MPa, *E*_unseeded, 21%_ = 1.06 MPa).

To better understand the impact of MSC seeding on 2D and 3D scaffolds, there was a need to level off the differences between unseeded samples by calculating the increase in UTS (Figure 5e) and the increase in *E* (Figure 5f). Increase in UTS showed statistically significant differences between 5% and 21% O_2_ in 3D scaffold (*p*_5–21%_ < 0.0001, Supplementary Table S8) and between 2D and 3D scaffold at 21% O_2_ (*p_2D–3D_* < 0.0001). Increase in *E* displayed statistically significant difference between 5% and 21% O_2_ in the 3D scaffold (*p*_5–21%_ = 0.034, Supplementary Table S9). Hence, MSC seeding mostly contributed to tensile properties of the 3D scaffold at 21% O_2_.

## 3. Discussion

To date, engineering in vitro bone grafts using multiple cell types, including MSCs, has focused on improving the tissue formation and functionality as the ultimate goal. However, there is a large gap between the O_2_ tension of osseous tissue where the graft is finally implanted and 21% O_2_ that is currently used for bone graft production. Thus, comparing the response of MSCs to these O_2_ tensions in detail may provide important insights to develop new approaches in which different O_2_ tensions are utilized for bone TE success. In the meantime, since bone TE applications either employ the direct seeding of cells onto scaffolds, thus a 2D approach [34] or embedding cells within 3D [35] scaffolds, we wanted to assess the roles of O_2_ tension and dimensionality simultaneously. To do so, we engineered biomimetic 2D and 3D scaffolds inspired by the in vivo bone lamellae and osteon, respectively.

In a previous publication, we reported that the MC-patterned 2D collagen/silk fibroin scaffold was biocompatible for ADSCs and permitted collagen type I and calcium deposition after 28 days in osteogenic medium [21]. These data imply that the collagen/silk fibroin scaffold enabled osteogenic differentiation in long term, making it an ideal substrate to investigate dimensionality.

The O_2_ level in the 3D scaffold was monitored to ensure its homogeneous distribution. The impaired diffusion of the O_2_ to the core and middle layers can lead to a sudden decrease in oxygenation, which can reduce cell viability and alter cell signaling, specifically for osteogenic and angiogenic signaling. It is particularly hard to maintain homogeneous O_2_ distribution within cell-loaded hydrogels, and hypoxic zones may arise even in the normoxic culture conditions [36]. For example, the O_2_ level in a synthetic hydrogel was measured around 1.5% after 7 days, and this low O_2_ availability adversely affected the MSC viability [37]. Our readings were above 1.5%, which suggesting that the required O_2_ level for MSC viability was preserved in the 3D collagen/silk fibroin scaffold. Within our 3D scaffold, we measured similar pO_2_ levels at the core and between the layers, suggesting the unconstrained movement of O_2_ throughout the 3D scaffold attributed to the layered structure. This property was deemed critical since low O_2_ availability could decrease even further directing MSC differentiation.

The MC patterns were degradation resistant, as SEM images demonstrated. Having long-lasting topographies is a desirable trait for the guidance of MSCs and neo-ECM throughout the prolonged culture conditions. MSCs were aligned on the MC trajectory. However, the variation in the MSC morphology was observed on the 3D scaffold surface only in the cases of 5% and 12% O_2_ tensions. At 5% O_2_, cell clumps existed, and this change might indicate the retention of stem cell phenotype since circular cell clumps are typically seen in undifferentiated embryonic cell culture [38]. Individual spindle-shaped cells at 12% O_2_ might show the MSC differentiation. In brief, these differences at 5% and 12% O_2_ tensions suggested that the introduction of the third dimension via 3D scaffold affected the MSC behavior.

Tissue culture plates or dishes are used as 2D culture systems to test O_2_ tension on cell behavior [10,11,14,15,39,40,41,42,43]. Wagegg et al. [14] and Hung et al. [43] demonstrated that 1% O_2_ promoted MSC osteogenesis at the gene-level and mineralization despite the contradictory findings at 2% O_2_ in a different study [40]. Interestingly, Raheja et al. reported no significant difference in osteogenic differentiation of MSCs between 5% and 21% O_2_ [11]. These variations may arise from the diversities in procurement and isolation protocols [43]. The shorter cell culture time used in gene analysis might also affect our results. Our data were in accordance with Wagegg et al. [14] and Hung et al. [43], and RUNX2 and OSX expressions on TCPS were lower at 21% O_2_. Additionally, 12% O_2_ seemed to promote MSC osteogenesis on TCPS. We also observed variations in the osteogenic and stemness transcript levels between TCPS and 2D scaffold. Thus, the gene expression analysis demonstrated that TCPS was not an appropriate 2D control. Thereby, we recommend using same substance in 2D and 3D environments to prevent misleading outcomes during the assessment of dimensionality.

Concerning dimensionality, the round and softer nature of the 3D substrate is mostly preferred over the 2D for reflecting the geometric complexity of the native cell microenvironment [44,45,46,47,48,49,50]. Signal accumulation and cell functions are regulated closer to native tissue conditions in the 3D environment [51,52]. The 3D geometry allows better spatial arrangement in cell polarity [48], integrin ligands [49], and adjacent cells. As a consequence, we anticipated elevated osteogenesis in 3D. Lower RUNX2 and higher OSX levels in 3D scaffold suggested the temporal differences in osteogenic marker expression between 2D and 3D scaffold geometries. This observation was in parallel with a study employed the ADSCs [15]. Our profile indicated that peak expression occurred earlier in 3D, whereas 2D was slower in osteogenic differentiation indicated by higher RUNX2 and lower OSX expression. Likewise, embryonic stem cells [17], human bone precursor cells [18], osteoblasts [18], and periodontal ligament fibroblasts [19] were shown to produce more late osteogenic markers such as collagen type I, ALP, osteonectin, osteocalcin, and osteopontin upon transition to the third dimension. The positive drive of third dimension for MSC osteogenesis was also supported with lower stemness levels compared to the 2D scaffold. Imamura et al. employed 3D spheroids and demonstrated reduced stemness relative to MSC monolayer after osteogenesis induction [53]. Similar to OSX, VEGFA expression elevated in 3D scaffold compared to 2D at every O_2_ tension tested. Higher VEGF production by MSCs is crucial to recruit endothelial cells for neovascularization at the implant site [54,55]. At this point, we highlighted the importance of cell-matrix interactions on MSC performance using a 3D scaffold made of concentric layers.

Following the dimensionality, outcomes of the O_2_ tension were examined in 2D and 3D scaffolds. Generally, on the 2D scaffold, all markers tested had a negative correlation to O_2_ tension. High expression of markers was observed at 5% O_2_, and the lowest values were observed at 21% O_2_. In turn, in the 3D scaffold, the highest expression levels were at 21% O_2_, and the lowest were at 12% O_2_. Similarly, He et al. reported that not the 1% and 5% O_2_ but atmospheric O_2_ tension led to the highest ADSC osteogenic activity in 3D poly(lactide-coglycolide) (PLG) scaffold [15]. Put together, it can be suggested that 12% O_2_ had the lower contribution to osteogenesis, stemness, and angiogenesis in both scaffolds. Interestingly, at 21% O_2_, stemness and osteogenesis markers were pronounced in 3D scaffold. The reason might be attributed to the early timing of day 3 for commitment and/or the presence of heterogeneous phenotypes within the MSC population.

Mechanical evaluation of the end product is extremely critical in bone TE. To our knowledge, there are no published studies showing the effects of dimensionality and O_2_ tension on the tensile properties of bone grafts. Here, O_2_ led to variation in the tensile strength of unseeded 2D scaffold. The decrease in UTS and E at 5% O_2_ can be inferred as the high degradation of the 2D scaffold with low O_2_ availability. The low dissolved O_2_ of 5% can increase the acidity of cell culture medium [56], which in turn can facilitate the hydrolysis of blend material. On the other hand, for the unseeded 3D specimens, 5% O_2_ had no adverse effect on the UTS and E as the similar values with 21% O_2_ pointed out. Thus, we suggested that the stability or protection of the collagen/silk fibroin material by the rolling process can retain strength of 3D scaffold similar to that at 21% O_2_ even at the unfavorable conditions of 5% O_2_. 

As mentioned before, osteogenic markers on the 2D scaffold were more pronounced at 5% O_2_. Hence, we anticipated higher tensile properties at 5% O_2_ due to expectedly more neo-ECM secretion on the surface of 2D scaffold. However, according to results, the MSC seeding significantly influenced the tensile properties at 21% O_2_, not at 5%. The reason for conflicting results might be the aggravated hydrolysis at 5% O_2_. We suspected that adverse conditions at 5% O_2_ probably led to low tensile properties, even if MSCs had secreted substantial ECM on 2D scaffold.

Further analysis by increase in UTS and increase in E data showed that the simultaneous contribution of 3D and 21% O_2_ on tensile properties was very effective. However, at 21% O_2_, 2D was not a supportive matrix as much as 3D for the MSCs to deposit their ECM on. We compared our data with the native bone tissue strength previously reported. Casari et al. tensile tested the dry state of ovine cortical bone in lamellar scale [57]. The average UTS at the axial direction of osteon was 350 MPa, while the transverse value was 130 MPa. However, UTS values were detected much higher than the osteon’s actual wet state due to toughening of dried specimen [57]. Another study was held by Seto et al. using the fibrolamellar calf bone, which consisted of several layers. UTS of wet fibrolamellar bone was determined as 75.9 MPa at the uniaxial direction to fibrils [58]. Perpendicular measurement was 4.9 MPa [58], and this value is close to the UTS of MSC-seeded 3D scaffold at 21% O_2_ (1.82 MPa).

Lastly, the microchannels and layered structure used in the scaffold design can give new insight into the development of 3D scaffolds sustaining sufficient O_2_ for cell metabolism both at tissue culture normoxia and physiological oxygen conditions. Enabling this feature in bulk materials can be problematic on the contrary. Enhanced expression of key markers for neo-bone tissue formation and improved tensile properties at 21% O_2_ can also be important for the progress of bone TE, because nonphysiological culture conditions employed in MSC-seeded 3D scaffolds may actually be favorable for bone and vascular gene expressions, stronger ECM deposition, and preservation of neo-ECM/scaffold’s tensile strength.

## 4. Materials and Methods

### 4.1. Production of Templates

Parallel MCs of 5 µm ridge width and depth, and 10 µm groove width were formed on the surface of the silicon wafer (Supplementary Figure S1a). The MC-patterned area (1 cm × 1 cm) was positioned at the center of the silicon wafer surface (1.5 cm × 1.7 cm). Microfabrication of the topography was carried out using the reactive ion etching (RIE) method at Bilkent University (Ankara, Turkey). The negative replica of micropatterns was transferred to poly(dimethylsiloxane) (PDMS) (Supplementary Figure S1b). To prepare the PDMS, the prepolymer and the curing agent were mixed in a 10:1 ratio (Sylgard 184 Elastomer Kit, Dow Corning, Midland, MI, USA), and then the polymer was crosslinked at 70 °C for 3 h. 

While one PDMS piece was employed for the 2D scaffold production (Supplementary Figure S2a), five crosslinked PDMS pieces were joined end-to-end to form the mold for 3D scaffold production (Supplementary Figure S2b). A single PDMS piece had the surface dimensions of 1.5 cm × 1.7 cm (Supplementary Figure S1c). To assemble the mold of the long film strip, the patterned side of the PDMS pieces were attached upside down to a glass slide surface using a double-sided tape after cutting the smooth surfaces between the MC-patterned area of each piece (Supplementary Figure S1d). The glass slide including the PDMS pieces was stuck by a double-sided tape onto a glass petri plate. To connect and stabilize the PDMS pieces, melted paraffin wax was poured into the petri dish by covering the half-height of the PDMS pieces. The wax solidified at room temperature, and then PDMS:curing agent mix was added into the petri dish until PDMS pieces were fully submerged. To completely stabilize the connected parts, PDMS was left to polymerize at room temperature. To clean the micropatterned side of the PDMS pieces from wax, the wax part was liquefied by heating, and the patterned surface was washed with isopropyl alcohol. The final mold had a surface area of 1.5 cm × 5.7 cm.

### 4.2. Isolation of Collagen Type I

Collagen type I was isolated from Sprague Dawley rat tails. Purification of the collagen was carried out according to a protocol described previously [22]. Rat tail tendons were dissolved in acetic acid (0.5 M, 4 °C). The solution was filtered with glass wool and dialyzed (molecular weight cut-off: 10kDa) against phosphate buffer (24 mM, pH 7.2). The pellet was centrifuged and dissolved in acetic acid (0.15 M). Protein was precipitated by adding NaCl (5%). On the following day, the precipitate was centrifuged and dissolved in acetic acid. After dialysis and centrifugation, pellets were sterilized in ethanol (70% (*v/v*)). Lastly, collagen was lyophilized (Labconco Freezone 6) for long-term storage.

### 4.3. Isolation of Silk Fibroin

Silk fibroin was purified from the silk threads of *Bombyx mori*, as explained in an earlier study [59]. Na_2_CO_3_ solution (0.02 M) was boiled, and silk threads (12.5 g) were washed for 30 min in the boiling Na_2_CO_3_ solution. Then, the threads were washed with distilled water and dried at 37 °C. Fibers were dissolved in LiBr solution (9.3 M, 60 °C), and the solution was filtered with filter paper to eliminate the remaining particles. The solution was dialyzed (molecular weight cut-off: 10kDa) against water for 3 days. After that, the solution of silk fibroin was lyophilized for long-term storage.

### 4.4. Preparation of 2D and 3D Collagen/Silk Fibroin Scaffolds

The use of unpatterned samples was excluded in the current experimental design because the positive effect of same MC patterns on the alignment and osteogenesis of ADSCs with respect to unpatterned controls was reported earlier [21]. The MC-patterned 2D scaffold (Supplementary Figure S1e) and the long strip of MC-patterned film (Supplementary Figure S1f) were prepared on the PDMS molds by the solvent casting of collagen/silk fibroin solution. The solution of collagen type I (1.6% (*w/v*)) and silk fibroin (0.8% (*w/v*)) was prepared in HFIP and then transferred to the normal-sized and the long PDMS templates (250 µL cm^−2^). The solution was dried at room temperature to allow film formation on the PDMS templates. Films were removed from the PDMS surfaces with a tweezer.

To induce the formation of β-sheets in the silk fibroin structure, dried films were subjected to methanol (90% (*v/v*)) for 1 h. Instead of using cytotoxic molecules such as glutaraldehyde [60], a highly biocompatible carbodiimide chemistry was employed to form amide bonds between collagen and silk fibroin. Crosslinking with EDC (Thermo Scientific, Waltham, MA, USA) and NHS (Sigma, St. Louis, MO, USA) allowed a further improvement in the stability of biodegradable 2D and 3D scaffolds after the methanol exposure. The crosslinking solution was prepared using EDC (12.5 mM) and NHS (5.2 mM) in methanol solution (90% (*v/v*)). Collagen/silk fibroin films were incubated in the crosslinking solution overnight. The next day, films were washed with distilled water, and they were air-dried at room temperature. After drying, films were stored in a desiccator. To prepare the 3D scaffold that was used in the imaging experiments, a long strip of MC-patterned film was wetted in distilled water. It was then converted to a roll using a Teflon rod that had a 2 mm diameter. To prevent its opening, the end of the film was sealed with cyanoacrylate glue. The thickness of the 2D and 3D scaffolds were measured with a micrometer after submerging them into cell culture medium.

### 4.5. Stereomicroscopy Imaging of Scaffolds

The overall morphology of the crosslinked 2D and 3D scaffolds was imaged with a stereomicroscope. Before the imaging, the 2D scaffold and the long film strip were wetted with distilled water. The reason for imaging at the wet state was to observe the changes in the scaffold structure after they were transferred into the cell culture medium.

### 4.6. Fluorescence Microscopy and CLSM

Cross-section of the 3D scaffold was observed using the 5(6)-carboxyfluorescein (Sigma-Aldrich) conjugated collagen/silk fibroin. The concentration of the fluorophore was 6.4 mM in the collagen/silk fibroin solution. After the addition of 5(6)-carboxyfluorescein, scaffold production, methanol stabilization, and crosslinking with EDC/NHS were carried out as prementioned. Utilization of the EDC/NHS in the crosslinking procedure was giving the advantage of amide bond formation between the carboxyl group of the fluorophore and the amine groups of collagen and silk fibroin. Before the imaging, the 3D scaffold was wetted with distilled water. The roll was cut in half with scissors and examined in the wet state with fluorescence microscopy (Zeiss, Jena, Germany). For the detailed imaging of the layers, CLSM (Leica DM2500) was utilized at the higher magnification. The distance between the layers was evaluated using the ImageJ software (NIH) and measured by randomly selecting the spacings between the rolled layers (*n* = 18).

### 4.7. Surface and Pattern Size Examination by SEM

The unseeded 2D and 3D scaffolds were examined with QUANTA 400F field emission SEM at an operating voltage of 10 kV. To section the 3D scaffold, the wetted roll was cut in half with scissors in the vertical direction to the MCs. Next, 2D and 3D scaffolds were placed on the stubs in the half-wet state. After air-drying the scaffolds, they were sputter-coated with Au-Pd (3 nm). The dimensions of the micropatterns were measured with ImageJ software.

### 4.8. Isolation of Human MSCs

Human MSCs were isolated from the bone marrow aspirations harvested from the iliac crest of patients that undergone total hip replacement surgery (Royal National Orthopaedic Hospital, Stanmore, UK) and stored at −80 °C until use. Written and signed patient consent and ethical committee approval from NHS Health Research Authority (National Research Ethical Committee, London) were obtained (REC reference number 07/Q0506/10). The characterization and multipotent differentiation potential of the used MSCs were demonstrated by Reissis et al. [61]. Cells between the passages of two to five were used for the experiments. For thawing the cells, the cell culture medium at 37 °C was added to the vial (Corning) and then transferred to a universal tube filled with 10 mL of cell culture medium. After centrifugation at 2000 rpm for 5 minutes, the supernatant was discarded, and cells were resuspended with the medium. The cell suspension was transferred to T125 flasks (Corning). MSCs were cultured in high glucose DMEM (Sigma Aldrich, St. Louis, MO, USA) supplemented with 10% FBS (First Link), 1% penicillin-streptomycin (Gibco, Carlsbad, CA, USA), and 5 ng/mL bFGF (Gibco). Flasks were placed in an incubator at 37 °C (95% relative humidity, 5% CO_2_, 21% O_2_; Sanyo, Osaka, Japan), and medium was changed every other day until the 80–90% confluency. MSCs were passaged using 0.1% trypsin-EDTA (Gibco).

### 4.9. Seeding of MSCs and Maintenance of Tissue-Engineered Scaffolds

2D scaffolds and long films were sterilized in ethanol (70% (*v/v*)) for 2 h and washed three times with phosphate-buffered saline (PBS, 10 mM, pH: 7.4). After air-drying the scaffolds, cells were detached from the flask surface with 0.1% trypsin-EDTA. Growth medium was added for enzyme inactivation, and MSCs were centrifuged at 3000 rpm for 5 minutes. Next, 5 × 10^4^ cells were seeded to the middle of 2D scaffold surface. To maintain the same MSC density on the micropatterned surface of long film, 25 × 10^4^ cells were seeded. A drop of suspension with 5 × 10^4^ cells was added similar to 2D scaffold to each five part of the long film strip one by one. The 3D scaffold was set by rolling and sealing the end of the long film after cell seeding. 2D and 3D scaffolds were placed in 12-well plates (Corning) and osteogenic medium (high glucose DMEM medium containing 10% FBS, 1% penicillin-streptomycin, 100 nM dexamethasone (Sigma Aldrich), 10 mM β-glycerophosphate (Sigma Aldrich), and 50 µg/mL ascorbate 2-phosphate (Sigma Aldrich)) was added.

For the real-time RT-PCR and mechanical test experiments, the number of seeded cells was increased four-fold. This change was made to obtain enough RNA levels and adequate neo-ECM deposition to be able to monitor the differences in gene expression and tensile properties of the scaffolds, respectively. At this part, 2 × 10^5^ MSCs/well were plated to TCPS (6-well plate, Corning), and also the same type of plate was employed during the culture of 2D and 3D scaffolds. After adding osteogenic medium, all of the samples were cultured in a CO_2_ incubator (Sanyo). At the beginning of the cell culture, the incubator (95% relative humidity, 5% CO_2_, 37 °C) was adjusted to either of the 5%, 12%, and 21% O_2_ tensions. The osteogenic medium was renewed every other day.

### 4.10. Measurement of Oxygen Levels inside the 3D Scaffold

To monitor the O_2_ concentration inside of the MSC-seeded 3D scaffold, fiber-optic O_2_ probes (Oxford Optronix) were placed into the core and between the layers at day 0. The position of the measuring tip was adjusted to the halfway along the long axis of 3D scaffold. The molecular oxygen-sensitive tip was 280 μm in diameter. The measurement was carried out by tracking the change in the probe luminescence. In principle, at the higher local O_2_ level, more luminescence is quenched. 

After MSC seeding and preparation of the 3D scaffold by rolling, O_2_ probe setup and measurement were performed in compliance with Cheema et al. [62]. Equipment and 3D scaffolds were kept at 37 °C for up to 1 hour before the O_2_ monitoring because O_2_ solubility is sensitive to temperature fluctuations. Sampling was initiated after the equilibration of O_2_ to the desired level inside the incubator (5% and 21% O_2_). The pO_2_ was measured at a rate of 27 sampling/h for at least 9 h. Data were presented as pO_2_ (mm Hg) and also converted to the percentage level (1% O_2_ corresponds to 7.6 mm Hg).

### 4.11. SEM

After 3 days of cell culture, samples were washed with cacodylate buffer (0.1 M, pH: 7.4) and fixed with 2.5% glutaraldehyde (in cacodylate buffer) for 2 h at room temperature. Scaffolds were rewashed, and 3D scaffolds were unrolled. Cells were stained with 1% osmium tetroxide (in cacodylate buffer) for 1 h. Samples were washed and then dehydrated with a series of graded ethanol (10 min 50%, 70%, 80%, 95% (twice), and 15 min 100%). Samples were mounted on aluminum stubs and sputter-coated with Au-Pd before analysis. Micrographs were taken with high-resolution field emission SEM (Jeol) at an operating voltage of 3 kV.

### 4.12. Real-Time RT-PCR 

At day 3, Trizol (Life Technologies, Carlsbad, CA, USA) was added on TCPS, 2D, and 3D scaffolds (*n* = 3). Cells on the TCPS were detached with the cell scraper (Corning). Scaffolds were chopped with a scalpel blade, and then all samples were kept at −80 °C. After thawing the specimens, the equal volume of ethanol (100% (*v/v*)) (Sigma) was added to the suspension. For the RNA extraction, Direct-zol RNA Miniprep (Zymo Research, Irvine, CA, USA) was used according to the manufacturer’s protocol. RNA was quantified employing Nanodrop (Thermo Scientific, Waltham, MA, USA). Total RNA of each sample was reverse-transcribed to complementary DNA (cDNA) with high-capacity cDNA reverse-transcription kit (without RNase inhibitor, Applied Biosystems, Foster City, CA, USA). Target cDNA levels were quantified using the iTaq^™^ Universal SYBR® Green Supermix (Biorad, Hercules, CA, USA).

Hypoxanthine phosphoribosyltransferase 1 (HPRT1) and 18S ribosomal RNA (18S rRNA) were employed as housekeeping genes. RUNX2, OSX, BST1, CD90, and VEGFA were the genes studied. Information about the primers was given in Supplementary Table S10. Real-time RT-PCR was performed with a CFX96 Real-Time System (Biorad). The conditions were 2 min at 50 °C and 10 min at 95 °C in the holding stage, then in the cycling stage 50 cycles at 95 °C for 15 s and at 60 °C for 1 min. Measurements were normalized relative to the housekeeping gene using the ΔΔ_CT_ method. Since primers were purchased from different brands, HPRT1 and 18S rRNA were used as housekeeping genes of the markers from the same brand during the calculations. HPRT1 (Biorad) was employed for RUNX2 (Biorad), BST1 (Biorad), and VEGFA (Biorad) genes. In addition, 18S rRNA (Qiagen, Germantown, MD, USA) was utilized for OSX (Qiagen) and CD90 (Qiagen) genes. The expression of each marker was presented relative to the TCPS sample at 21% O_2_. The value of the TCPS sample at 21% O_2_ was assigned as 1, and the rest of the calculations were performed accordingly.

### 4.13. Tensile Testing

The tensile properties of the unseeded and MSC-seeded scaffolds were examined after 35 days of cell culture to give enough time to cells for ECM deposition to the extent that can alter the tensile strength. Here, the aligned cells on the MC topography are expected to deposit ECM at the same trajectory [22]. Therefore, this approach helps to obtain strong ECM in the uniaxial direction as in the native lamella and osteon.

Unseeded 2D and 3D collagen/silk fibroin scaffolds were incubated at the same conditions with MSC-seeded scaffolds. Tensile testing was performed using an Instron 3366 uniaxial testing machine with a 10 N load cell (Instron Engineering Corporation). Samples (*n* = 3) were tested at room temperature by applying the force parallel to the MC axis. Before the test, none of the samples were fixed chemically. To prepare the 3D scaffolds for the test, they were opened, and sections were cut. Next, 2D scaffolds and sections from the 3D scaffolds were placed on a rubber base tool. A parallel double-blade was utilized to uniformly cut the section width to 4 mm and bring them into a standard size. The final film strips had a 15 mm length at the MC direction. The ends of each strip were held by grips that reduced the apparent distance between clamp faces to 10 mm. The setup was subsequently transferred inside a Perspex chamber filled with PBS to prevent material dehydration.

After completing the setup, a preload (0.01 N, rate: 1 mm/min) was applied to correct a possible loose layout between the clamps. Preload was in the uniaxial tension mode, and no data were recorded in the meantime. After preload, the uniaxial tension was exerted with an elongation rate of 0.04 mm/min (equal to 1.0%/min strain rate). The test was continued until specimen failure. 

The measurements of applied force (F), cross-sectional area (A), initial length (l), and elongation (Δl) were used to calculate the stress (σ = F/A), strain (ε = Δl/l), UTS (maximum σ), and *E* (σ/ε). To determine the increase in UTS, the average UTS value of unseeded scaffolds was subtracted from the UTS of MSC-seeded samples. The same method was applied to calculate the increase in *E*.

### 4.14. Statistical Analysis

All data were expressed as the mean value ± standard deviation. The data were analyzed with statistical analysis software (SPSS 25.0, IBM). Significant differences in gene expression levels, increase in UTS, and increase in *E* values were determined with two-way ANOVA followed by Bonferroni post hoc test. During UTS and *E* assessment, the groups were analyzed using three-way ANOVA followed by Bonferroni post hoc test. Statistical differences were presented as * *p* ≤ 0.05, ** *p* ≤ 0.01, *** *p* ≤ 0.001, and **** *p* ≤ 0.0001.

## 5. Conclusions

There is still room for advancements in protocol optimization for MSC isolation and ex vivo preparation of bone tissue-engineered products for clinical use. These practices should be well established and standardized [63]. Raw materials from human and animal sources can have contamination and disease transmission problems. Additionally, the removal of living cells from these materials is a must to continue with the scaffold processing [64]. Materials that do not evoke immune response in patients can lower the costs and ease with regulatory issues. Once these issues are solved, storage, quality control, logistics, and shipping are moderately settled topics [64].

Following the developments in the field, we presented a way to harness the powers of dimensionality and O_2_ tension together. We produced a 3D biomimetic collagen/silk fibroin scaffold that enabled adequate perfusion of O_2_. The layered scaffold recapitulated the bone osteon and promoted MSC osteogenesis and angiogenesis, as well as enhanced the tensile properties at 21% O_2_. At tested O_2_ tensions, the use of the 2D scaffold was indeed limiting the expression of vascular and early osteogenic markers and a barrier to obtaining satisfactory tensile strength. For a better understanding in the dimensionality concept, the necessity for the same material usage in 2D and 3D environments was also shown. Our findings can also have broader implications concerning the future of MSC-seeded bone graft production.

## Data Availability

MDPI Research Data Policies.

## Supplementary Materials

The following are available online at https://www.mdpi.com/article/10.3390/ijms22084010/s1, Figure S1: specifications of the silicon wafer, PDMS replicas, and films, Figure S2: PDMS replicas had the negative prints of the MCs, Table S1: pairwise comparison of RUNX2 expression, Table S2: pairwise comparison of OSX expression, Table S3: pairwise comparison of BST1 expression, Table S4: pairwise comparison of CD90 expression, Table S5: pairwise comparison of VEGFA expression, Table S6: pairwise comparison of UTS values, Table S7: pairwise comparison of *E* values, Table S8: pairwise comparison of increase in UTS values, Table S9: pairwise comparison of increase in *E* values, Table S10: information about the target genes for real-time RT-PCR.
