# Supplementary material for: Evaluating Oxygen Tensions Related to Bone Marrow and Matrix for MSC Differentiation in 2D and 3D Biomimetic Lamellar Scaffolds
Source: Int J Mol Sci. 2021 Apr 13;22(8):4010. doi: 10.3390/ijms22084010 (PMC8068918; doi:10.3390/ijms22084010)
Supplement: Supplementary file 1 [file ijms-22-04010-s001.pdf]

Supplementary Information

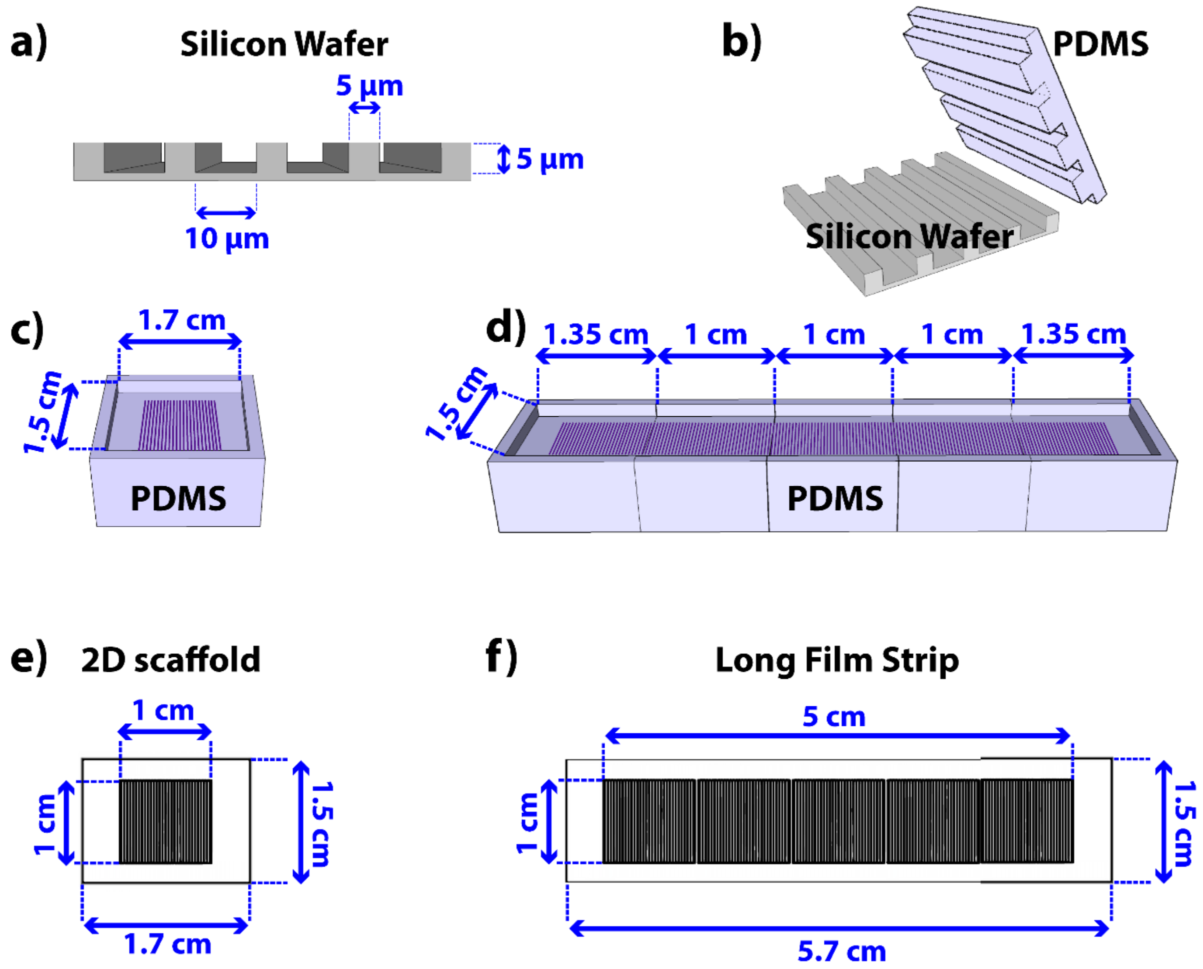

Figure S1. Specifications of the silicon wafer, PDMS replicas, and films. (**a**) Design of the microchannels (MCs) features on the silicon wafer. (**b**) PDMS was manufactured, and it had a negative copy of the MCs on its surface. Dimensions of the PDMS pieces were shown (**c**) for single and (**d**) combined layout. Dimensions of the collagen/silk fibroin films and their patterned regions. (**e**) 2D scaffold. (**f**) Long film strip that was used in 3D scaffold preparation.

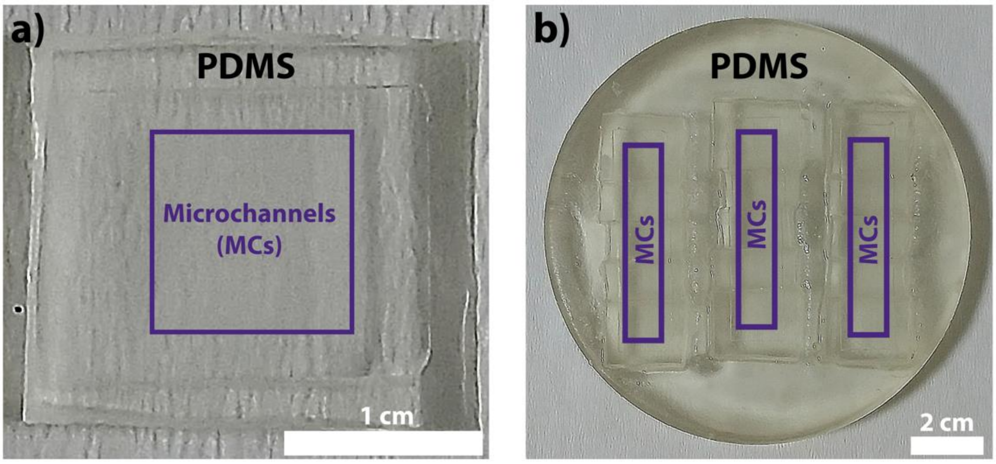

Figure S2: PDMS replicas had the negative prints of the MCs. They were prepared for the production of (**a**) 2D scaffold as the single piece and (**b**) long film strip as the combined five pieces.

Table S1: Pairwise comparison of RUNX2 expression.

Group	Pairs	Statistical difference
TCPS	5% - 12%	*
TCPS	5% - 21%	*
TCPS	12% - 21%	****
2D	5% - 21%	*
3D	12% - 21%	**
5%	TCPS - 3D	***
5%	2D - 3D	***
12%	TCPS - 2D	****
12%	TCPS - 3D	****
12%	2D - 3D	**

* $p \leq 0.05$, ** $p \leq 0.01$, *** $p \leq 0.001$, and **** $p \leq 0.0001$

Table S2: Pairwise comparison of OSX expression.

Group	Pairs	Statistical difference
3D	5% - 21%	****
3D	12% - 21%	****
5%	TCPS - 3D	**
5%	2D - 3D	**
12%	TCPS - 3D	*
12%	2D - 3D	*
21%	TCPS - 3D	****
21%	2D - 3D	****

* $p \leq 0.05$, ** $p \leq 0.01$, *** $p \leq 0.001$, and **** $p \leq 0.0001$

Table S3: Pairwise comparison of BST1 expression.

Group	Pairs	Statistical difference
TCPS	5% - 21%	**
2D	5% - 12%	****
2D	5% - 21%	****
5%	TCPS - 2D	***
5%	2D - 3D	**

* $p \leq 0.05$, ** $p \leq 0.01$, *** $p \leq 0.001$, and **** $p \leq 0.0001$

Table S4: Pairwise comparison of CD90 expression.

Group	Pairs	Statistical difference
TCPS	5% - 12%	**
TCPS	5% - 21%	****
TCPS	12% - 21%	*
3D	5% - 21%	**
3D	12% - 21%	****
5%	TCPS - 3D	****
5%	2D - 3D	****
12%	TCPS - 2D	**
12%	TCPS - 3D	**
12%	2D - 3D	****
21%	TCPS - 2D	****
21%	TCPS - 3D	***

* $p \leq 0.05$, ** $p \leq 0.01$, *** $p \leq 0.001$, and **** $p \leq 0.0001$

Table S5: Pairwise comparison of VEGFA expression.

Group	Pairs	Statistical difference
3D	5% - 12%	****
3D	5% - 21%	****
3D	12% - 21%	****
5%	TCPS - 3D	****
5%	2D - 3D	****
12%	TCPS - 3D	***
12%	2D - 3D	***
21%	TCPS - 3D	****
21%	2D - 3D	****

* $p \leq 0.05$, ** $p \leq 0.01$, *** $p \leq 0.001$, and **** $p \leq 0.0001$

Table S6: Pairwise comparison of UTS values.

Group	Subgroup	Pairs	Statistical difference
2D	Unseeded	5% - 21%	**
2D	MSC seeded	5% - 21%	**
3D	MSC seeded	5% - 21%	****
21%	MSC seeded	2D - 3D	**
21%	3D	Unseeded - MSC seeded	****

* $p \leq 0.05$, ** $p \leq 0.01$, *** $p \leq 0.001$, and **** $p \leq 0.0001$

Table S7: Pairwise comparison of *E* values.

Group	Subgroup	Pairs	Statistical difference
2D	Unseeded	5% - 21%	*
2D	MSC seeded	5% - 21%	****
3D	MSC seeded	5% - 21%	**
21%	2D	Unseeded - MSC seeded	*
21%	3D	Unseeded - MSC seeded	**

* $p \leq 0.05$, ** $p \leq 0.01$, *** $p \leq 0.001$, and **** $p \leq 0.0001$

Table S8: Pairwise comparison of increase in UTS values.

Group	Pairs	Statistical difference
3D	5% - 21%	****
21%	2D - 3D	****

* $p \leq 0.05$, ** $p \leq 0.01$, *** $p \leq 0.001$, and **** $p \leq 0.0001$

Table S9: Pairwise comparison of increase in *E* values.

Group	Pairs	Statistical difference
3D	5% - 21%	*

* $p \leq 0.05$, ** $p \leq 0.01$, *** $p \leq 0.001$, and **** $p \leq 0.0001$

Table S10: Information about the target genes for real-time RT-PCR.

Gene	NCBI accession no.	Brand	Product code
HPRT1	NG 012329.1	Biorad	qHsaCID0016375
RUNX2	NG 008020.1	Biorad	qHsaCID0006726
BST1	NC 000004.11	Biorad	qHsaCID0013947
VEGFA	NG 008732.1	Biorad	qHsaCED0043454
18S rRNA	X03205.1	Qiagen	QT00199367
OSX	NM 152860.1	Qiagen	QT00213514
CD90	NM 006288.4	Qiagen	QT00023569
